# Plant leaf disease recognition based on improved SinGAN and improved ResNet34

**DOI:** 10.3389/frai.2024.1414274

**Published:** 2024-06-24

**Authors:** Jiaojiao Chen, Haiyang Hu, Jianping Yang

**Affiliations:** College of Big Data, Yunnan Agricultural University, Kunming, China

**Keywords:** plant leaf disease identification, SinGAN, autoencoder, convolutional block attention module, ResNet34

## Abstract

The identification of plant leaf diseases is crucial in precision agriculture, playing a pivotal role in advancing the modernization of agriculture. Timely detection and diagnosis of leaf diseases for preventive measures significantly contribute to enhancing both the quantity and quality of agricultural products, thereby fostering the in-depth development of precision agriculture. However, despite the rapid development of research on plant leaf disease identification, it still faces challenges such as insufficient agricultural datasets and the problem of deep learning-based disease identification models having numerous training parameters and insufficient accuracy. This paper proposes a plant leaf disease identification method based on improved SinGAN and improved ResNet34 to address the aforementioned issues. Firstly, an improved SinGAN called Reconstruction-Based Single Image Generation Network (ReSinGN) is proposed for image enhancement. This network accelerates model training speed by using an autoencoder to replace the GAN in the SinGAN and incorporates a Convolutional Block Attention Module (CBAM) into the autoencoder to more accurately capture important features and structural information in the images. Random pixel Shuffling are introduced in ReSinGN to enable the model to learn richer data representations, further enhancing the quality of generated images. Secondly, an improved ResNet34 is proposed for plant leaf disease identification. This involves adding CBAM modules to the ResNet34 to alleviate the limitations of parameter sharing, replacing the ReLU activation function with LeakyReLU activation function to address the problem of neuron death, and utilizing transfer learning-based training methods to accelerate network training speed. This paper takes tomato leaf diseases as the experimental subject, and the experimental results demonstrate that: (1) ReSinGN generates high-quality images at least 44.6 times faster in training speed compared to SinGAN. (2) The Tenengrad score of images generated by the ReSinGN model is 67.3, which is improved by 30.2 compared to the SinGAN, resulting in clearer images. (3) ReSinGN model with random pixel Shuffling outperforms SinGAN in both image clarity and distortion, achieving the optimal balance between image clarity and distortion. (4) The improved ResNet34 achieved an average recognition accuracy, recognition precision, recognition accuracy (redundant as it’s similar to precision), recall, and F1 score of 98.57, 96.57, 98.68, 97.7, and 98.17%, respectively, for tomato leaf disease identification. Compared to the original ResNet34, this represents enhancements of 3.65, 4.66, 0.88, 4.1, and 2.47%, respectively.

## 1 Introduction

Precision agriculture (Chen et al., 2021) is a product of the rapid development of artificial intelligence and Internet of Things technology, playing an important role in promoting China’s agriculture toward modernization. Plant leaf disease identification, as one of the research areas in precision agriculture, has a significant impact on the development of the planting industry (Liao et al., 2018). Timely and effective detection and diagnosis of leaf diseases, along with preventive measures, can significantly increase crop yield and quality, further driving the advancement of precision agriculture.

In recent years, with the rapid development of computer vision technology, deep learning methods have been widely applied in crop disease identification. Wang et al. (2024) proposed a tomato leaf disease detection method based on attention mechanisms and multi-scale feature fusion. By incorporating CBAM into the backbone network to enhance lesion feature extraction and reduce environmental interference, they constructed the BiRepGFPN module to fuse shallow features for improved small lesion localization capability, which was then applied to the YOLOv6 model replacing PAFPN, effectively fusing deep semantic and shallow spatial information. The model achieved respective improvements of 2.3, 4.0, 3.1, and 2.7% in accuracy, recall, F1 score, and mAP on a tomato leaf disease dataset. Gonzalez-Huitron et al. (2021), addressing the classification of tomato leaf diseases, combined transfer learning with four lightweight models. They conducted both quantitative and qualitative evaluations through quality metrics and saliency maps, and developed a GUI tool adaptable to different devices. Hu and Fang (2022) achieved automatic identification of tea leaf diseases with small sample sizes by integrating the MergeModel with leaf disease image segmentation and weight initialization techniques, and further augmenting the dataset through generating new training samples using SinGAN. Compared to existing methods, the proposed approach demonstrates higher accuracy in the recognition of tea leaf diseases with limited samples. Amri et al. (2024) introduced a new deep learning model, MIV-PlantNet, tailored for Saudi Arabia’s diverse plant population, which integrated the advantages of MobileNet, Inception, and VGG architectures, achieving a 99% accuracy rate, 96% precision, and 98% F1 score, demonstrating superior performance. Alkanan and Gulzar (2024) enhanced the MobileNetV2 model by incorporating multi-level optimizations and combining various model tuning techniques, applying the refined model to corn disease classification tasks. Comparative analysis against state-of-the-art models revealed that the improved MobileNetV2 surpassed its counterparts in terms of accuracy, recall, F1 score, and overall accuracy. Zeng et al. (2024)proposed the DIC-Transformer model, which first detects disease areas using Faster R-CNN combined with Swin Transformer and generates disease image feature vectors, then employs a Transformer to generate image descriptions, enhancing subsequent classifier decoder performance through weighted fusion of text features with image feature vectors. Experiments showed that DIC-Transformer outperforms other comparative models in both classification and description generation tasks. Wu et al. (2024) applied an improved Mask R-CNN method to the image segmentation task of soybean rust pathogens. This method initially replaces the original backbone of Mask R-CNN with Res2net to hierarchically split residual connections within a single residual block and combines FPG to reinforce feature extraction capabilities. Furthermore, it adopts the CIoU loss function in the bounding box regression prediction stage to expedite model convergence and cater to the precise classification needs of high-density spore images. Compared to the original Mask R-CNN algorithm, the improved version saw respective enhancements of 6.4, 12.3, and 2.2% in detection and segmentation mAP and accuracy.

This paper presents a plant leaf disease identification method based on an improved SinGAN (Shaham et al., 2019) and an enhanced ResNet34 (He et al., 2016), with key contributions outlined as follows:

1. Accelerated Model Training: Replacing GAN in SinGAN with an autoencoder shifts the training objective from unconditional image generation to image reconstruction, leading to a faster training process for the model.
2. Accelerated Model Training: Replacing GAN in SinGAN with an autoencoder shifts the training objective from unconditional image generation to image reconstruction, leading to a faster training process for the model.
3. Accelerated Model Training: Replacing GAN in SinGAN with an autoencoder shifts the training objective from unconditional image generation to image reconstruction, leading to a faster training process for the model.
4. Accelerated Model Training: Replacing GAN in SinGAN with an autoencoder shifts the training objective from unconditional image generation to image reconstruction, leading to a faster training process for the model.
5. Accelerated Model Training: Replacing GAN in SinGAN with an autoencoder shifts the training objective from unconditional image generation to image reconstruction, leading to a faster training process for the model.
6. Accelerated Model Training: Replacing GAN in SinGAN with an autoencoder shifts the training objective from unconditional image generation to image reconstruction, leading to a faster training process for the model.

**Figure 1:**
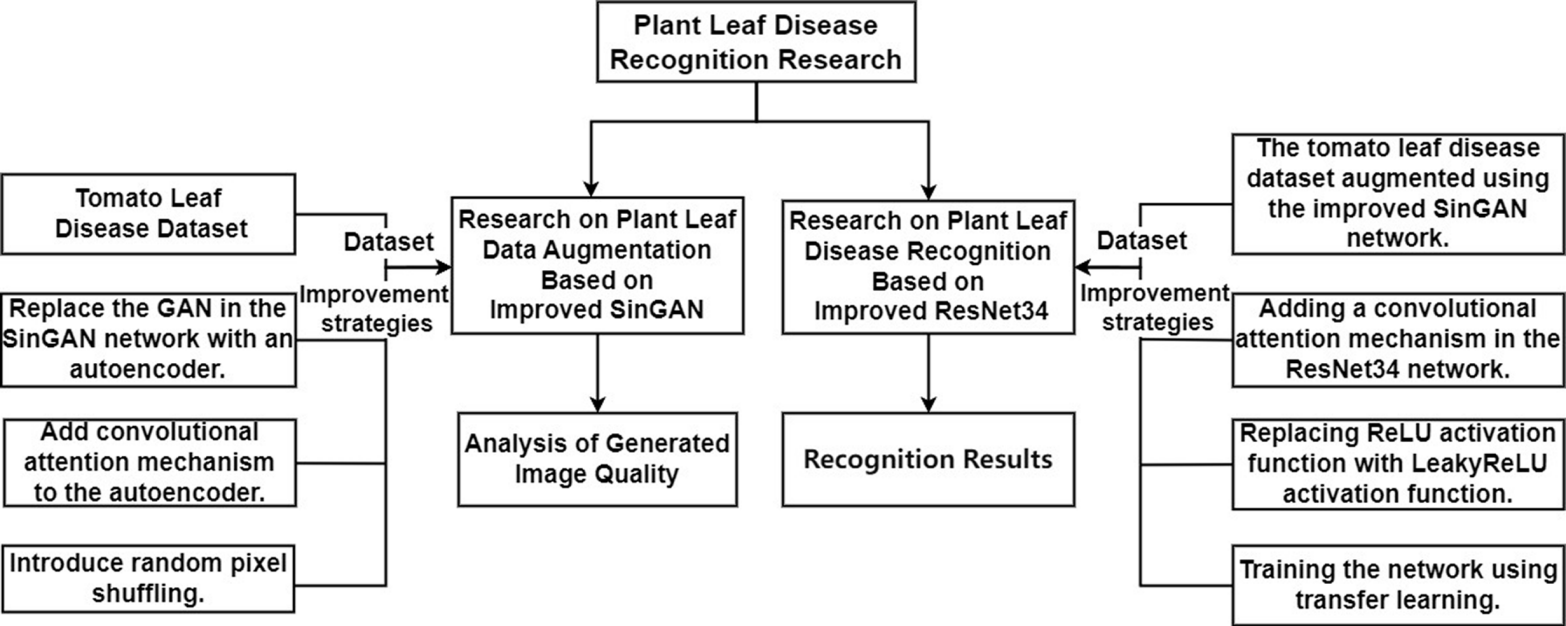
The technology roadmap.

The technical roadmap of this paper is illustrated in Figure 1.

## 2 Materials and methods

### 2.1 SinGAN

**Figure 2:**
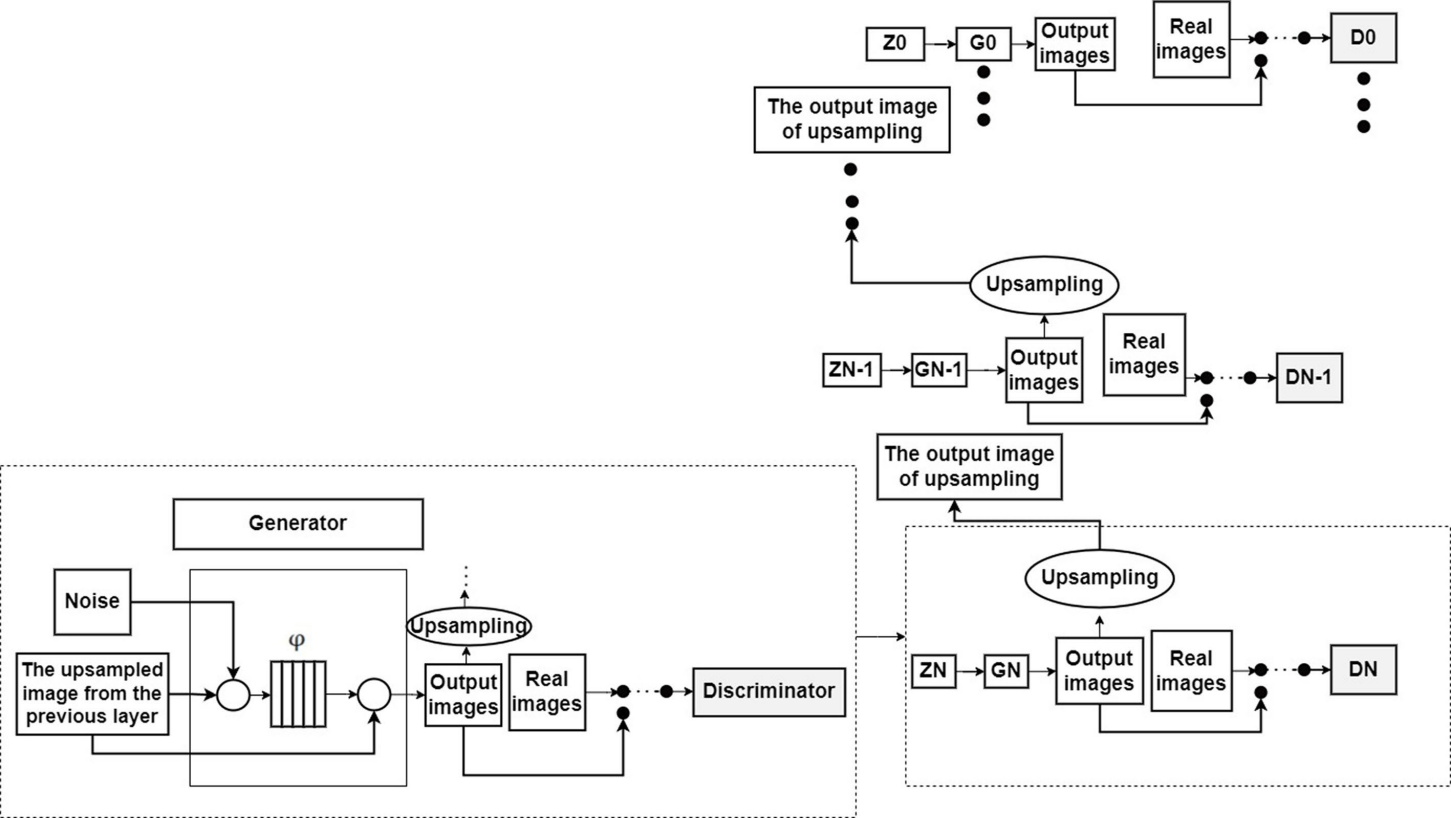
The basic structure of the SinGAN.

Shaham et al. (2019) proposed the SinGAN model, which is a non-conditional generative model that learns from a single image to generate images from noise. Like conventional GANs, SinGAN aims to fit the distribution of real data by progressively capturing data relationships within the samples. As shown in Figure 2, the output images of the $GAN_{n}$ layer are upsampled by cubic interpolation to the next scale resolution, then fed along with noise $Z_{n-1}$ into the generator of the $GAN_{n-1}$ layer. The images generated through training with convolutional neural networks are combined with the images $X_{n}$ generated at $GAN_{n}$ layer to obtain fake images $X_{n-1}$ at that resolution. Subsequently, these fake images and real images are input together into the discriminator to determine if they are real images at the current scale. Through multiple rounds of training, SinGAN can generate high-quality images $X_{n-1}$ . From $GAN_{n}$ to $GAN_{0}$ , N + 1 scale structures are progressively trained to learn the internal data distribution of a single image at different resolutions.

### 2.2 Data augmentation method based on improved SinGAN

SinGAN is a single-image training and generation model based on Generative Adversarial Networks (GANs), which circumvents the need for a large number of training samples, requiring only a single image to achieve diverse image generation. However, despite training with only a single image, SinGAN involves iterative training at multiple scales, which may require longer training times and higher computational resources. Additionally, since SinGAN is trained on a single image, the generated images may suffer from distortion issues. This paper proposes ReSinGN to circumvent the above-mentioned issues, and its model architecture follows that of SinGAN. ReSinGN is a reconstruction-based single-image generative network trained for general image generation on a single natural image. The network replaces the GAN in SinGAN with an autoencoder incorporating the CBAM, introduces random pixel Shuffling within the model, and trains the model in a cascaded multi-scale and progressively growing manner to capture information at different scales of a single image. Experimental results demonstrate that ReSinGN has shorter training times compared to SinGAN and generates higher-quality tomato leaf disease images. Furthermore, ReSinGN outperforms the SinGAN in terms of distortion scores.

#### 2.2.1 Reconstruction-based single image generation network

**Figure 3:**
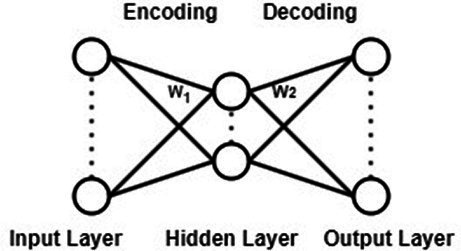
Autoencoder model structure.

Autoencoder (Hinton and Salakhutdinov, 2006) is a data-driven, unsupervised learning neural network model used for extracting data features. Its structure is depicted in Figure 3. It can be observed from Figure 3 that the autoencoder is divided into an encoding stage and a decoding stage. Its objective is to capture essential factors representing the input data through the intermediate hidden layer encoding, while the output layer’s result is solely used to assess whether the autoencoder can reconstruct the original data. By computing the reconstruction error and utilizing the backpropagation algorithm for parameter optimization, the autoencoder aims to achieve reconstruction error within a given range, thereby obtaining the features of the input data.

The aim of this paper is to train a model for generating high-quality images from a single natural image more quickly. While SinGAN addresses the similar problem, its learning objective is complex, requiring excessive time for model training. Inspired by Yoo and Chen (2021), this paper replaces the GAN in the SinGAN with an autoencoder, forming a Reconstruction-Based Single Image Generation Network (ReSinGN). The model transitions from learning unconditional image generation to learning image reconstruction, making the training objective simpler.

#### 2.2.2 Adding convolutional block attention module

**Figure 4:**
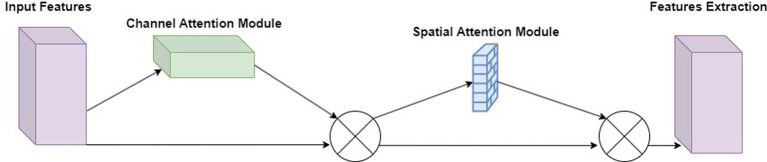
Connection between channel module and spatial module in CNN.

The Convolutional Block Attention Module (Woo et al., 2018) consists of two sub-modules: the Channel Attention Module and the Spatial Attention Module. By adaptively refining intermediate feature maps through CBAM in each convolutional block of the deep network, feature representation capability can be enhanced, enabling the network to learn to focus on key information. As a lightweight attention module, CBAM can be embedded into almost all convolutional neural networks with minimal additional computational cost and parameter overhead. CBAM also supports end-to-end training with the base CNN, as shown in Figure 4.

**Figure 5:**
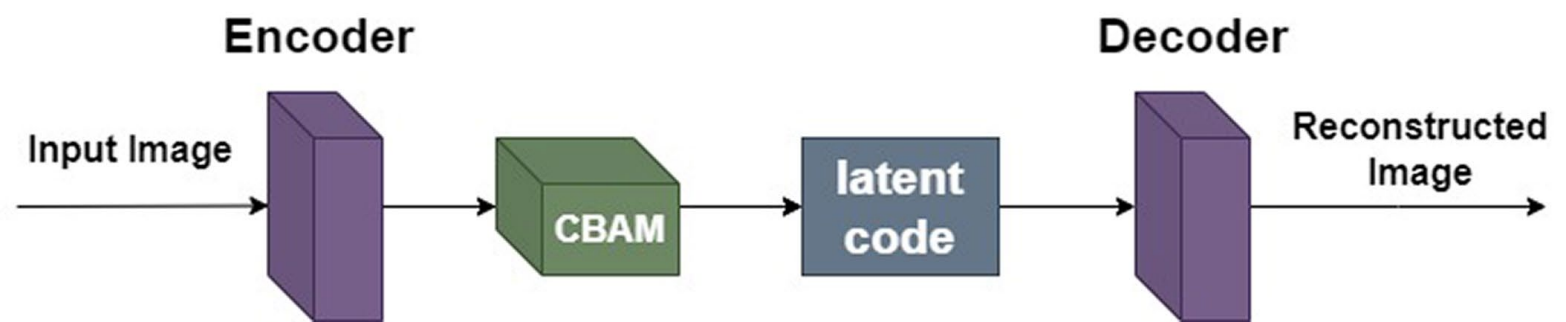
The network architecture diagram of ReSinGN integrated with CBAM module.

Due to the limitations of the autoencoder network structure, it may not effectively capture important local and global features in the image, resulting in distortion and blurriness in the reconstruction results. To enhance the model’s perception of local and global features in images and improve reconstruction quality, this paper incorporates the attention mechanism module CBAM (Convolutional Block Attention Module) after the encoder of the autoencoder, as shown in Figure 5. Adding the CBAM module to the encoder part allows it to flexibly select and strengthen important features in the input data, enabling the model to more accurately capture important details and structural information in the image, thereby improving the reconstruction quality of the autoencoder and enhancing the overall performance of the model.

#### 2.2.3 Multi-scale cascaded learning

**Figure 6:**
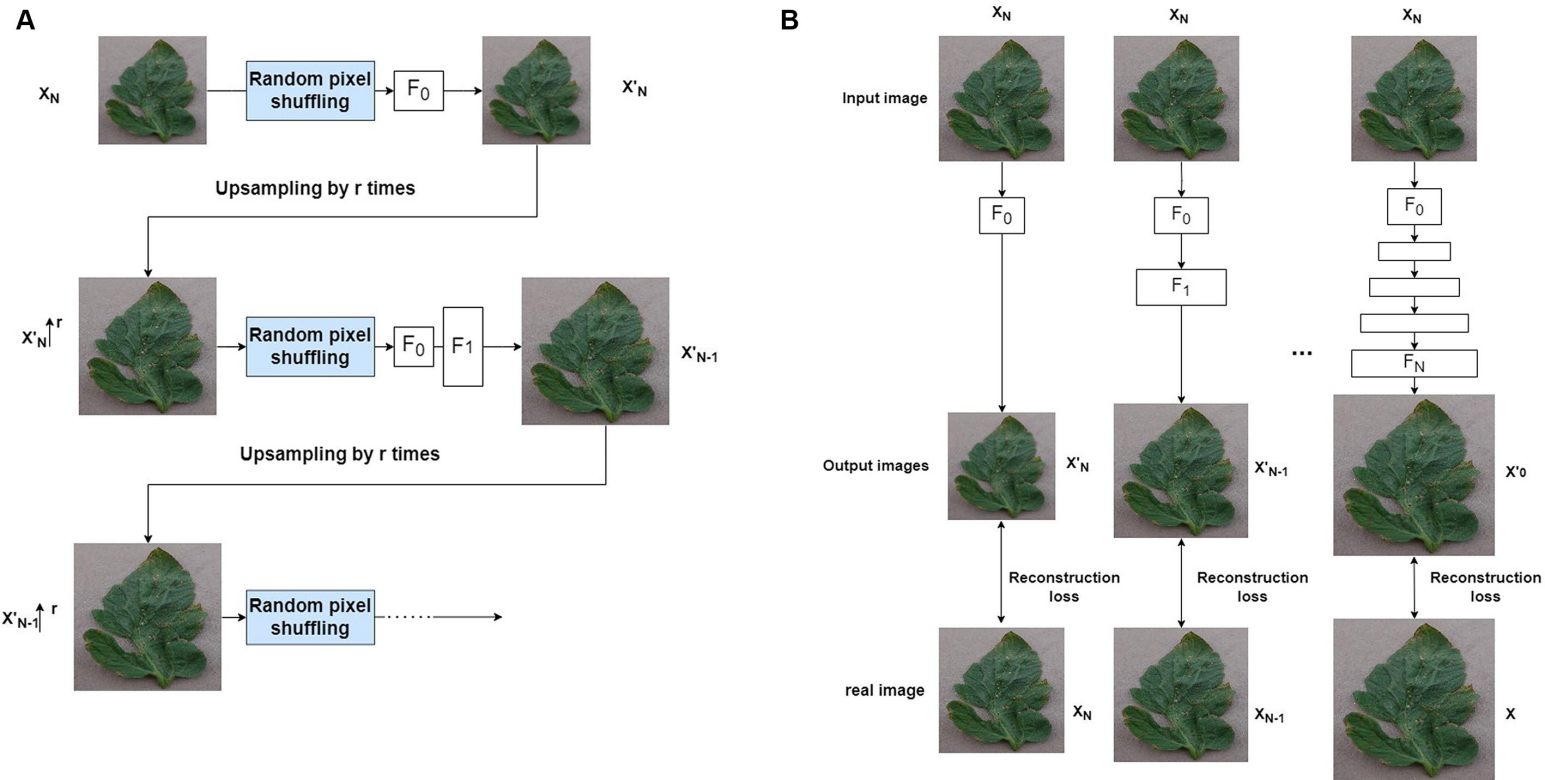
**(A)** Cascading multi-scale training. **(B)** Step by step learning training.

A natural image typically contains different structures at various scales. In order to successfully learn these cross-scale visual attributes, similar to SinGAN, ReSinGN learns how to refine a downsampled training image in a cascaded multi-scale manner (Figure 6A), to learn image visual attributes at different scales. The model consists of multiple networks, each responsible for refinement at each scale. The output of each network is upsampled and fed into the next finer-scale network. At coarser scales, the model learns more to refine the overall structure, while at finer scales, it learns more to refine details and textures. In this way, the entire model can reconstruct high-resolution images that are closer to the original image.

To train the multi-scale network, this paper adopts a progressively growing learning approach (Karras et al., 2017) to train the ReSinGN model (Figure 6B). This is an advanced multi-scale learning framework widely used in many image synthesis methods (Aigner and Körner, 2018; Karras et al., 2019; Zhang et al., 2019), including SinGAN. This method starts from the coarsest scale and progressively trains multiple networks, freezing the previously trained networks when continuing to train the next scale network. This approach addresses simpler problems one by one, thereby reducing the training difficulty. Specifically, the model first downsamples the training image to obtain a set of N + 1 real images at different scales $\{X_{N},X_{N-1},\ldots ,X_{1}$ }, where $X_{0}$ *=* X and the X is the original training image. Then, at each nth scale, the nth network $F_{n}$ is trained to reconstruct the image refined by one level. Therefore, let ${\hat{X}}_{n}=F_{n}\left({\hat{X}}_{n+1}{↑}^{r}\right)$ and ${\hat{X}}_{N}=F_{N}\left(X_{N}\right)$ represent the reconstructed image at the coarsest scale ( ${↑}^{r}$ represents upsampling by a factor of r ), the objective of this section is:


(1) $$\begin{array}{c} \min\\ F_{n} \end{array}L_{rec}\left(X_{n},{\hat{X}}_{n}\right)$$

$L_{rec}$ is the reconstruction loss. Once the training of a network is completed, this network is frozen, and a new network is added to train an image at a smaller scale.

#### 2.2.4 Random pixel shuffling

When the training objective is image reconstruction, the cascaded multi-scale learning method has certain limitations. This is because when the output at the coarsest scale exhibits limited diversity, it may show limited variations to the next network in the training samples. Consequently, in the progressive training process, the finer-scale networks are likely to learn simple or identity mappings, rather than more complex or diverse representations. Furthermore, since each network can only be trained with fixed outputs from previously frozen networks, the diversity at the input end of the network is zero. This approach restricts the network’s opportunities to learn from various variations, reducing the strength of its representation capabilities. Inspired by denoising autoencoders (Vincent et al., 2008), this paper effectively alleviates this issue by introducing a simple technique - random pixel shuffling, specifically, random permutation of the input image pixels (Figure 6A). This method introduces randomness, making the mapping of the autoencoder randomized. This process allows the autoencoder to learn richer and more powerful data representations, even when trained with reconstruction loss, thereby enabling it to generate new images. Additionally, the introduced random pixel transformations are used as tools to control the trade-off between super-resolution perception and distortion (Blau and Michaeli, 2018).

#### 2.2.5 Optimizer

This paper utilizes the Adam optimizer (Kingma, 2015) ( $\beta_{1}$ = 0.5, $\beta_{2}$ = 0.999) with a learning rate of 0.001. Equation (2) as the loss function for ReSinGN. The reconstruction loss employs the mean squared error (MSE) loss. However, the MSE loss tends to produce blurry images (Zhao et al., 2016). Therefore, a weighted sum of the mean squared error (MSE) loss and the Kullback–Leibler (KL) divergence is combined with the Structural Similarity (SSIM) loss (Wang et al., 2004). In Equation (1), $L_{rec}$ represents:


(2) $$\begin{array}{c} L_{rec\left(A,B\right)}=\mathrm{ms}e_{\mathrm{weight}}*MSE\left(A,B\right)+\left(1-\mathrm{ms}e_{\mathrm{weight}}\right)\\ {}*\;KL\left(A,B\right)+\left(1-\mathrm{SSIM}\left(A,B\right)\right) \end{array}$$

Where $\mathrm{ms}e_{\mathrm{weight}}$ is used to control the relative weight of the MSE and KL divergence losses in the overall loss calculation, and A and B are the original image and the reconstructed image, respectively.

#### 2.2.6 Model architecture

**Figure 7:**
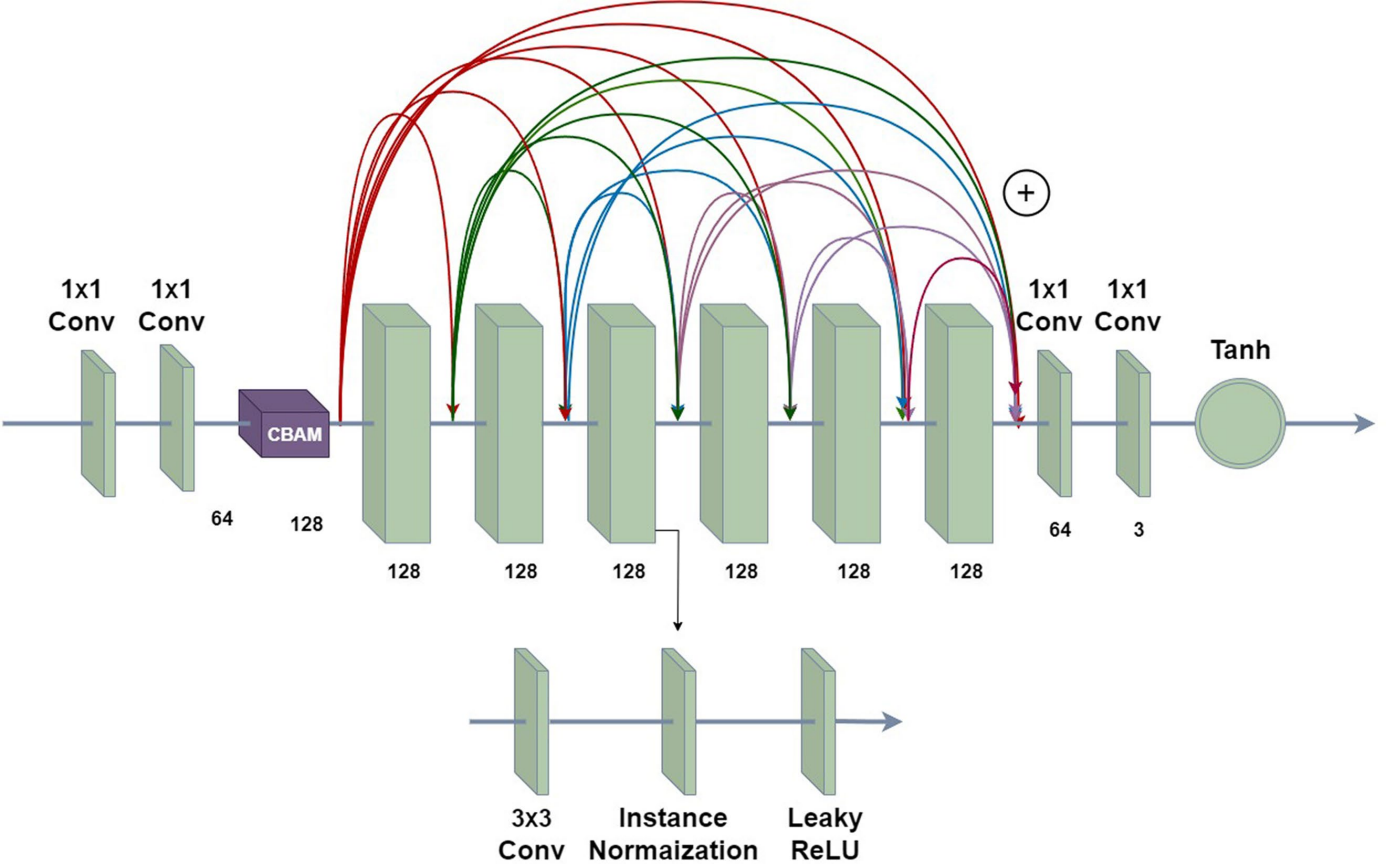
The network architecture of ReSinGN.

Figure 7 shows the network architecture of the ReSinGN model. The first two 1 × 1 convolutional layers encode the input data, mapping RGB images to the feature space, with a CBAM module added after the encoder. The last two 1 × 1 convolutional layers map the representations in the feature space back to the reconstructed input data. The middle six convolutional blocks are densely connected through residual operations (Huang et al., 2017), with each convolutional block consisting of a 3 × 3 convolutional layer, an instance normalization layer, and a LeakyReLU activation layer (with a negative slope of 0.2). The Tanh function is used to obtain the final output. No pooling or unpooling is used within the network, so the input and output of each network have the same spatial dimensions. Additionally, the ReSinGN network architecture is used for all networks at each scale.

### 2.3 Residual network model

**Figure 8:**
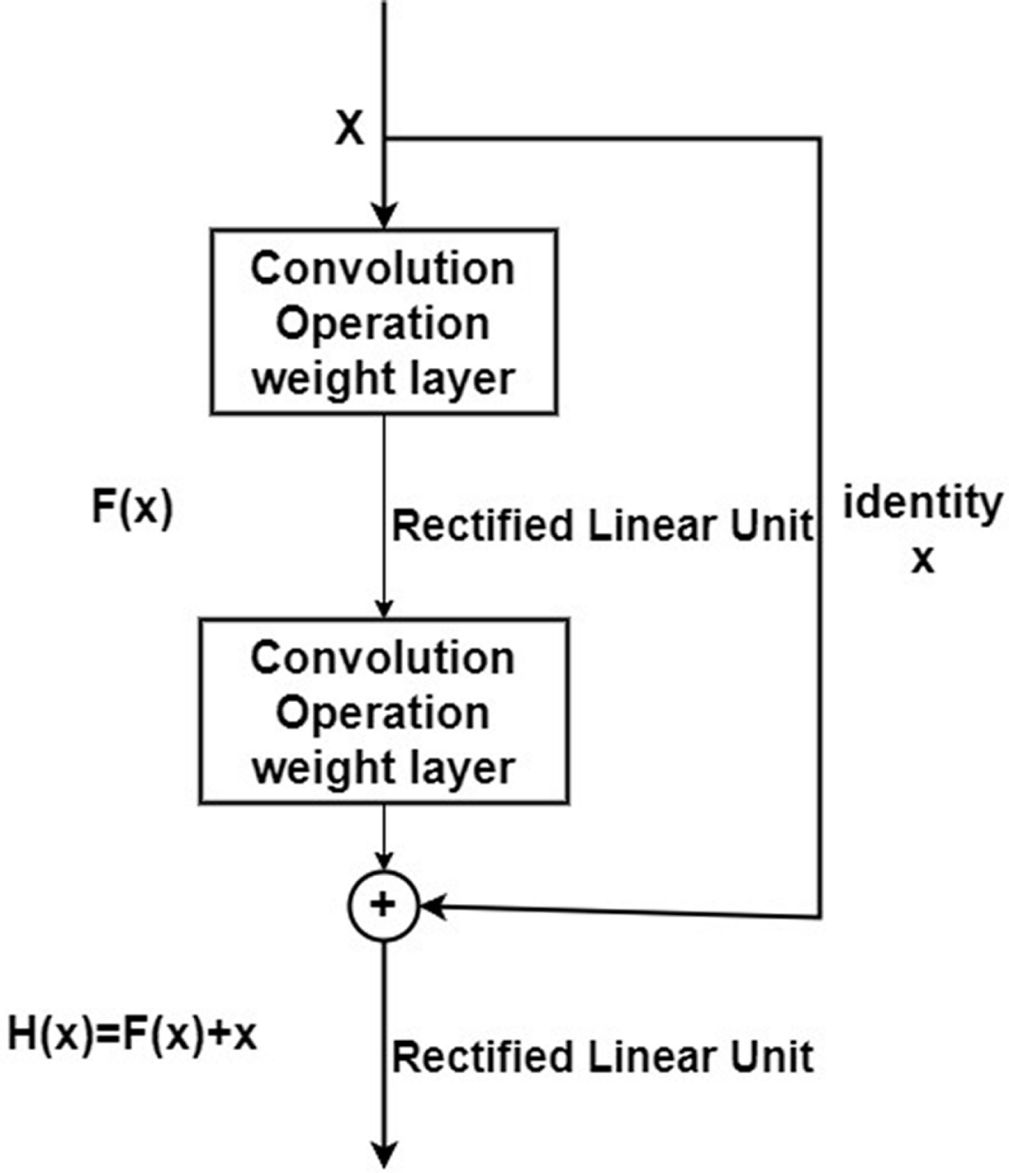
Residual block structure.

ResNet is a deeply influential neural network architecture introduced by the team led by Kaiming He in 2016. It directly increased the depth of neural networks to 152 layers in the ImageNet image classification competition. The model overwhelmingly won the championship in both image recognition and object detection tasks in ImageNet, and similarly excelled in object detection and image segmentation competitions on the COCO dataset. The introduction of ResNet has significant historical significance for the development of deep neural networks. Compared to traditional network architectures, ResNet introduced “shortcut connections” or “skip connections,” as shown in Figure 8.

ResNet introduces a “shortcut” connection before the ReLU activation function in the second layer, altering the input to the activation function from the original output H(x) = F(x) to H(x) = F(x) + x. In ResNet, this operation, which keeps the output equal to the input, is known as an identity mapping. The “identity” in the residual block structure in Figure 8 ensures the implementation of this identity mapping. With the introduction of identity, the identity mapping alters the direction of the network layer, that is F(x) + x → x. This improvement facilitates the direct propagation of data across multiple layers, ensuring that the model remains in an ideal state while aiding in the rapid convergence of the network.

In addressing the vanishing gradient problem, it is analyzed through Equation (3):


(3) $$X_{L}=X_{l}+\overset{L-1}{\underset{i=1}{\sum }}F\left(X_{i},W_{i}\right)$$

Where $X_{L}$ represents the characteristics of the L-th layer of the network, indicating that each unit $X_{l}$ in the shallow layers is augmented with a residual function mapping $\sum_{i=1}^{L-1}F\left(X_{i},W_{i}\right)$ , showing that the model exhibits residual characteristics within each unit. It can be observed that in the residual network, the output of lower-level residual modules can be determined by a higher-level layer.

For backpropagation, assuming the loss function is E, according to the chain rule of backpropagation, we can derive the gradient formula (4) for the residual network.


(4) $$\frac{\partial \varepsilon }{\partial X_{l}}=\frac{\partial \varepsilon }{\partial X_{L}}\frac{\partial X_{L}}{\partial X_{l}}=\frac{\partial \varepsilon }{\partial X_{L}}\left(1+\frac{\partial }{\partial X_{l}}\overset{L-1}{\underset{i=1}{\sum }}F\left(X_{i},W_{i}\right)\right)$$

Equation (4) consists of two parts: $\frac{\partial \varepsilon }{\partial X_{l}}$ that does not pass through the weight layers and $\frac{\partial \varepsilon }{\partial X_{L}}\left(1+\frac{\partial }{\partial X_{l}}\overset{L-1}{\underset{i=1}{\sum }}F\left(X_{i},W_{i}\right)\right)$ that passes through the weight layers, $\frac{\partial \varepsilon }{\partial X_{l}}$ ensures that the propagation can be directly fed back to any shallow layer $X_{l}$ , which is a consequence of the mapping in a typical multi-layer neural network. In the residual network, even if the gradients of the newly added multi-layer neural network are zero, an additional “1” is added during the gradient update process. This helps to avoid the problem of vanishing gradients, allowing the gradients from deeper layers to directly propagate back to the previous layer, enabling effective training of the parameters in shallow layers.

### 2.4 Plant leaf disease identification method based on improved ResNet34

For the medium-scale classification task of tomato leaf diseases, this study opts for ResNet34 as the foundational model, grounded in the following rationales: (1) On tasks of intermediate scale, ResNet34 strikingly balances high performance with computational efficiency, ensuring optimal resource utilization. (2) As a lighter variant within the ResNet family, ResNet34 is distinguished by its capability to yield excellent classification outcomes with reduced computational resources and shorter training durations, thereby making it a computationally frugal yet effective choice.

#### 2.4.1 Adding convolutional block attention module

**Figure 9:**
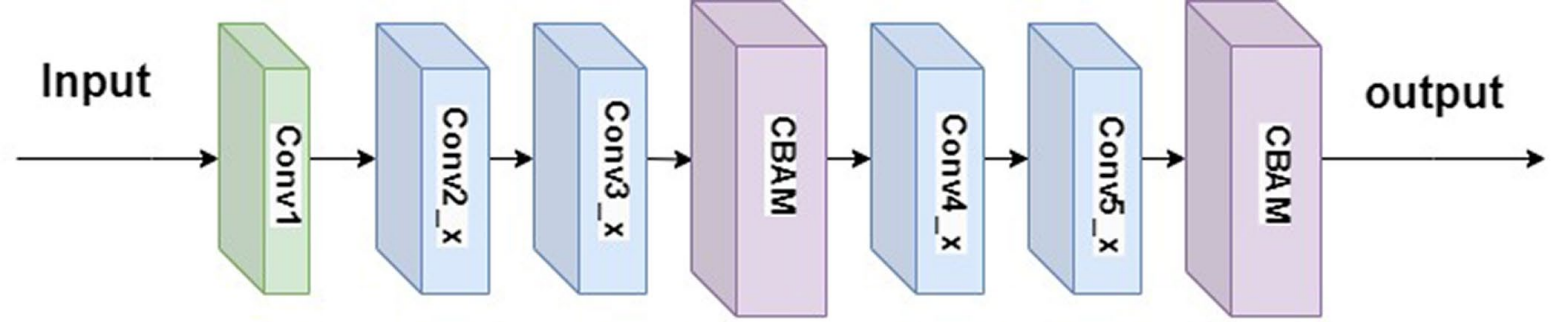
The ResNet34-CBAM network structure.

To address the issues of insufficient integration of local features and excessive parameter sharing in the ResNet34, this paper introduces the CBAM (Woo et al., 2018) into its architecture, resulting in ResNet34-CBAM. Simply adding the CBAM module directly to ResNet34 would alter the network structure, rendering the use of pre-trained parameters infeasible. Through experimentation, this study incorporated two CBAM modules into ResNet34, positioned after the second convolutional layer and the last convolutional layer, as illustrated in Figure 9. This combination exhibits higher weight coefficients at recognition points, thereby enhancing the classification performance of ResNet34.

**Figure 10:**
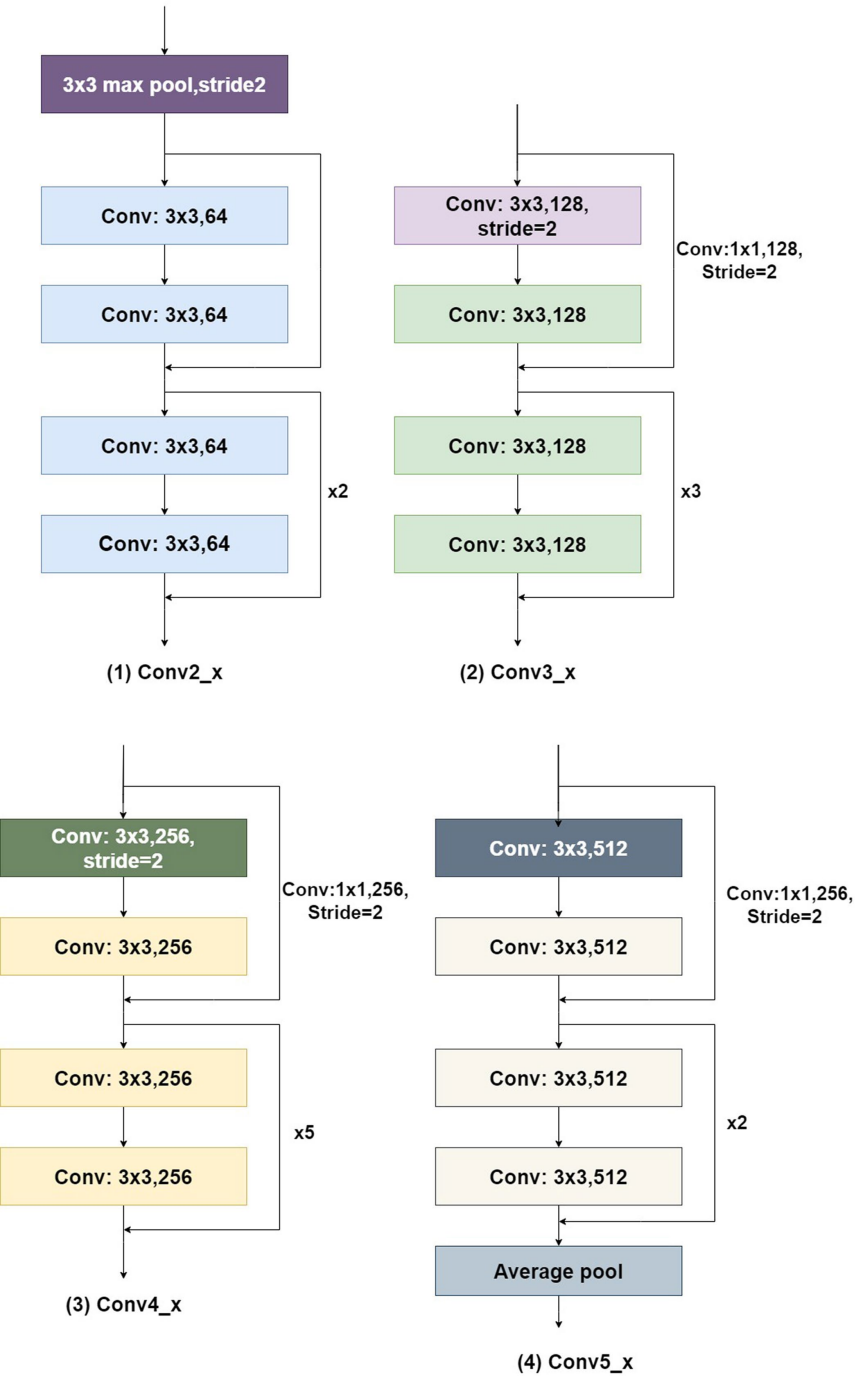
Residual structure in ResNet34.

The convolutional structure of Conv1 in Figure 9 is 7×7, with 64 channels and a stride of 2. The residual structures of Conv2_x, Conv3_x, Conv4_x, and Conv5_x are illustrated in Figure 10.

#### 2.4.2 LeakyReLU activation function

ReLU (Banerjee et al., 2019) is a commonly used activation function in neural networks, with its corresponding formula shown in Equation (5). It can be observed from Equation (5) that during the training process, the ReLU activation function only operates when the input variable x is positive. When the input variable x is negative, ReLU exhibits neuron death, resulting in the cessation of weight updates.


(5) $$\mathrm{ReLu}=\{\begin{array}{c} 1,x>0\\ 0,x\leq 0 \end{array}$$

LeakyReLU (Dubey and Jain, 2019) is very similar to the ReLU function, with the only difference lying in the negative part of the input. While ReLU sets the values of the negative part of the input to 0, LeakyReLU assigns negative values to the negative part of the input and has a small gradient. The formula for the LeakyReLU activation function is shown in Equation (6).


(6) $$\mathrm{LeakyReLu}=\{\begin{array}{c} x,x\geq 0\\ \mathrm{ax},x<0 \end{array}$$

In Equation (6), a usually takes a smaller value.

To address the issue of neuron death, this paper employs LeakyReLU instead of ReLU as the activation function in intermediate layers, forming ResNet34-LeakyReLU. During the training of the ResNet34-LeakyReLU model, the activation function in the negative region is more active. The advantage of using LeakyReLU lies in its ability to compute gradients even when its input values are less than zero during backpropagation. This characteristic not only prevents the phenomenon of node death during training but also enhances the adaptability and robustness of the model.

#### 2.4.3 Transfer learning

Transfer learning is a machine learning method, the core idea of which is to utilize existing knowledge to address problems in different but related domains (Zhuang et al., 2023). It aims to achieve knowledge transfer from one domain to another related domain. For convolutional neural networks, transfer learning involves successfully applying the “knowledge” trained on specific datasets to new domains.

Transfer learning can typically be applied to convolutional neural networks in two ways. The first approach involves using a pre-trained model with learned weights to extract features required for the new problem, essentially employing the pre-trained model as a feature extractor for the new problem. In this method, the features of interest are extracted from the output of the network preceding the last fully connected layer. The second approach is to fine-tune the network weights by training the network with new data. When adopting this method, it is necessary to adjust the number of nodes in the output layer to match the number of categories in the new problem. Additionally, regardless of the method chosen, the size of input data needs to be adjusted to match that of the pre-trained model. The specific transfer learning strategy should be determined based on the size and similarity between the target dataset and the original dataset. If the target dataset is very small and similar to the original dataset, using the pre-trained model as a feature extractor helps prevent overfitting; if there is a significant difference between them, fine-tuning is preferred.

**Figure 11:**
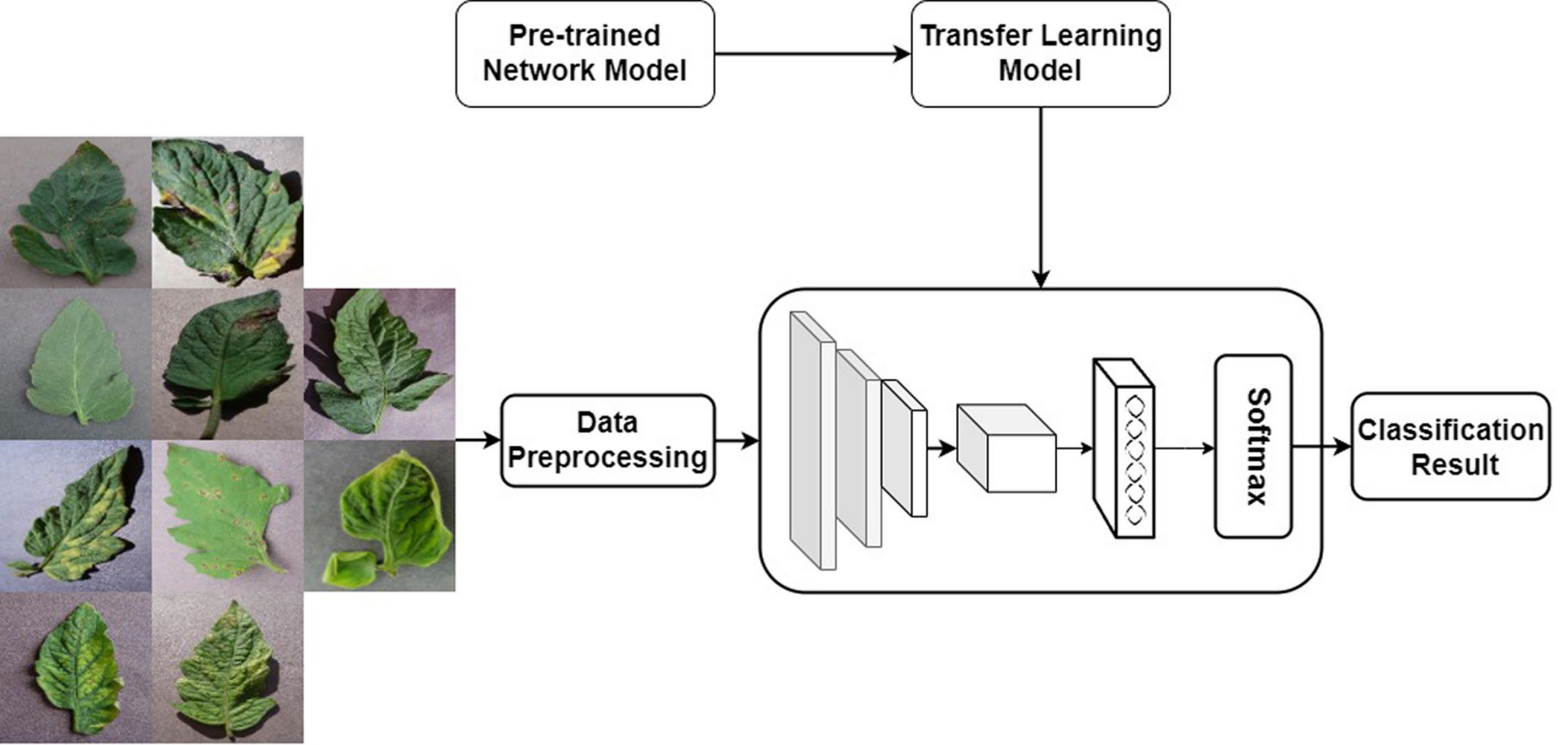
Transfer learning training process diagram.

Given that ResNet34 is a deep neural network primarily designed for extracting high-level features from large, complex datasets, and considering the relatively smaller size of the dataset employed in this study, which heightens the risk of overfitting, a pretrained model is utilized to extract features from the images. The trained ResNet34 is first used as a feature extractor, and then the extracted features are put into the Softmax classifier for classification training. The transfer learning process is shown in Figure 11.

### 2.5 Dataset

**Figure 12:**
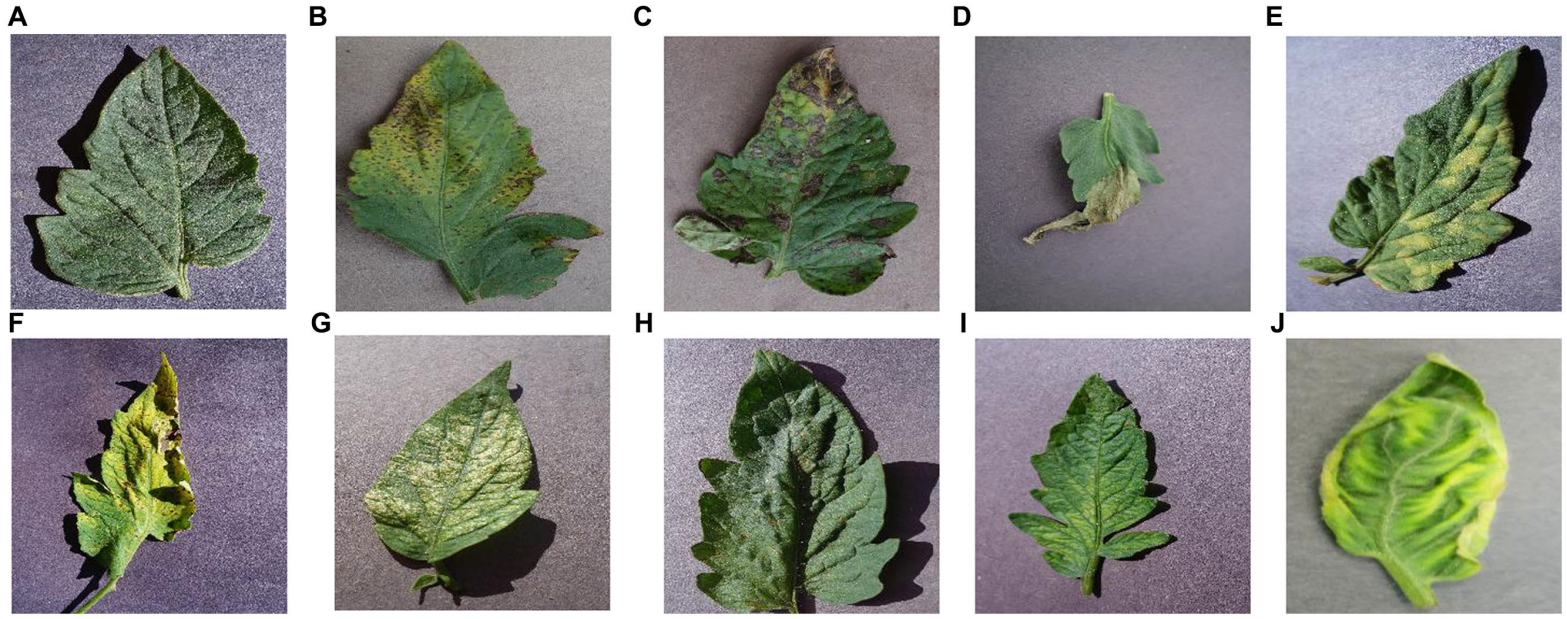
Each category of tomato leaf images. **(A)** healthy, **(B)** bacterial_spot, **(C)** early_blight, **(D)** late_blight, **(E)** leaf mold, **(F)** septoria leaf spot, **(G)** two spotted spider mite, **(H)** target spot, **(I)** mosaic virus, **(J)** yellow leaf curl virus.

The dataset used in this paper is from the publicly available dataset on “kaggle,” which contains 9 classes of tomato leaf diseases and class 1 of healthy leaves, with 700 images in each category for a total of 7,000 images. As shown in Figure 12.

### 2.6 Data augmentation

In order to enhance the model’s generalization ability and mitigate the risk of model overfitting, this subsection employs the ReSinGN method for data augmentation, expanding each category of tomato leaf diseases to 1,000 images. ReSinGN is trained with 2 scales, where the scale factor is set to 2, random pixel transformation percentage is set to 1e-2, and the learning rate is set to 0.001. The specific process of data augmentation is as follows: (1) Utilize a simple random sampling method to extract 300 images from each category of tomato leaves; (2) Employ the ReSinGN model to reconstruct the extracted 300 images, generating higher quality sample images; (3) Integrate the 3,000 images generated by ReSinGN back into the original dataset. Finally, the augmented dataset is divided into a training set, a validation set, and a testing set in a ratio of 7:2:1.

## 3 Experimental results

### 3.1 Experimental environment and parameter settings

All experiments in this paper were consistently conducted on a computer equipped with an NVIDIA RTX 3050 Ti GPU, utilizing Pytorch as the deep learning framework and Python as the programming language.

During the training process of ReSinGN, for fair comparison, this paper sets the scale factor r to approximately 4/3, with the minimum and maximum sizes being 25px and 250px respectively, following the same approach as SinGAN. The learning rate is set to 0.001, and bicubic interpolation is used for resampling. Regarding the number of iterations, since the training objective of ReSinGN is much simpler than unconditional image generation, significantly fewer iterations are required. It achieves optimal image generation results with only 500 iterations per scale, whereas SinGAN requires up to 4,000 iterations to achieve similar results.

During the model training process, all models share the same hyperparameters, as shown in Table 1.

**Table 1:** Model hyperparameter configuration.

Parameter name	Parameter value
Batch-size	16
Epochs	100
Optimizer	Adam
Learning rate	0.0001
Loss function	CrossEntropyLoss

### 3.2 Image evaluation metrics

The Tenengrad function (Zhao et al., 2004) is a commonly used method for evaluating image sharpness. It utilizes the information of image gradients to assess the clarity or focus sharpness of an image, where larger gradients typically indicate sharper images. The Tenengrad function employs Sobel operators to extract the gradients in both the horizontal and vertical directions, and then calculates the sum of their squares as the evaluation function. The specific process is as follows:

If the Sobel convolution kernel is denoted as $G_{x},G_{y}$ , then the gradient of the image at a point $\left(x,y\right)$ is given by:


(7) $$S_{\left(x,y\right)}=\sqrt{G_{x}*I\left(x,y\right)+G_{y}*I\left(x,y\right)}$$

The Tenengrad value of the image is defined as:


(8) $$\mathrm{Ten}=\frac{1}{n}*\underset{x}{\sum }\underset{y}{\sum }S{\left(x,y\right)}^{2}$$

The evaluation function $F\left(k\right)$ is:


(9) $$F\left(k\right)=\underset{x}{\sum }\underset{y}{\sum }{\left[G\left(x,y\right)\right]}^{2}\;\left(G\left(x,y\right)\right)>T$$

Where T is the given edge detection threshold.

RMSE (Root Mean Square Error) is a commonly used image quality evaluation metric. It assesses the quality of an image by calculating the difference in pixel values between the evaluated image and the original image. Generally, a lower RMSE value indicates a smaller difference between the image and the original image, indicating higher quality, while a higher RMSE value indicates a larger difference between the image and the original image, indicating lower quality. Its formula is as follows:


(10) $$\mathrm{RMSE}=\frac{1}{M\times N}*\overset{M}{\underset{i=1}{\sum }}\overset{N}{\underset{j=1}{\sum }}\left(f'\left(i,j\right)-f\left(i,j\right)\right)$$

Where $f'\left(i,j\right)$ represents the image to be evaluated, $f\left(i,j\right)$ represents the original image, M and N respectively represent the length and width of the image.

Peak Signal-to-Noise Ratio (PSNR) is an objective assessment method used to measure the level of distortion or noise in an image. It ranges from 0 to 40, with units in decibels (dB), where higher values indicate greater similarity between the reconstructed image and the original image. PSNR is defined as follows:


(11) $$\mathrm{PSNR}=10\log_{10}\frac{2^{n}-1}{MSE}$$

Where MSE (Mean Square Error) represents the mean square error between the original image and the reconstructed image:


(12) $$MSE=\frac{1}{H*W}\overset{H}{\underset{i=1}{\sum }}\overset{W}{\underset{j=1}{\sum }}{\left[s\left(i,j\right)-r\left(i,j\right)\right]}^{2}$$

Where n represents the number of bits per pixel, measured in bits, H and W respectively denote the height and width of the image, s and r respectively represent the reconstructed image and the original image.

SSIM (Wang et al., 2004) is a metric used to measure the similarity between two images. It assesses the distortion of images by comparing the structural changes in the data, thereby providing an objective quality assessment. The SSIM ranges from 0 to 1, where values closer to 1 indicate greater similarity between the images. SSIM measures image similarity in terms of brightness, contrast, and structure.


(13) $$l\left(x,y\right)=\frac{2u_{x}u_{y}+c_{1}}{u_{x}^{2}+u_{y}^{2}+c_{1}},c\left(x,y\right)=\frac{2\sigma_{x}\sigma_{y}+c_{2}}{\sigma_{x}^{2}+\sigma_{y}^{2}+c_{2}},s\left(x,y\right)=\frac{\sigma_{xy}+c_{3}}{\sigma_{x}\sigma_{y}+c_{3}}$$

(14) $$\sigma_{xy}=\frac{1}{N-1}\overset{N}{\underset{i=1}{\sum }}\left(x_{i}-u_{x}\right)\left(y_{i}-u_{y}\right)$$

Where l represents the brightness of the image, c denotes the contrast of the image, s represents the structure of the image, $\sigma_{x}$ signifies the structure of the image, $\sigma_{xy}$ represents the expectation, $\sigma_{y}$ is the standard deviation, $\sigma_{xy}$ indicates covariance, and $c_{1}$ , $c_{2}$ ,and $c_{3}$ are constants. Then, the formula for SSIM is given by:


(15) $$\mathrm{SSIM}\left(x,y\right)=l{\left(x,y\right)}^{\alpha }\cdot c{\left(x,y\right)}^{\beta }\cdot s{\left(x,y\right)}^{\gamma }$$

Where x represents the original image, and y denotes the reconstructed image. When the hyperparameter α,β,γ takes the value of 1 and c3=c22 , the structural similarity can be simplified to:


(16) $$\mathrm{SSIM}\left(x,y\right)=\frac{\left(2u_{x}u_{y}+c_{1}\right)\left(2\sigma_{xy}+c_{2}\right)}{\left(u_{x}^{2}+u_{y}^{2}+c_{1}\right)\left(\sigma_{x}^{2}+\sigma_{y}^{2}+c_{2}\right)}$$

### 3.3 Image classification criteria

This paper primarily utilizes accuracy, recall, precision, and F1 score Equations (17–20) to evaluate the performance of the network.


(17) $$\mathrm{Accuracy}=\frac{TP+TN}{TP+FP+TN+FN}$$

(18) $$\mathrm{Recall}=\frac{TP}{TP+FN}$$

(19) $$\mathrm{Precision}=\frac{TP}{TP+FP}$$

(20) $$F_{1}-\mathrm{score}=\frac{2\times \mathrm{Precision}\times \mathrm{Recall}}{\mathrm{Precision}+\mathrm{Recall}}$$

In the above formulas, Accuracy describes the ratio of correctly predicted instances among all predictions made by the algorithm. Recall describes the ratio of correctly predicted instances to all instances that should have been predicted correctly. Precision refers to the proportion of instances identified as positive that are actually positive samples. F1-score is a comprehensive evaluation metric. TP (True Positives) and FN (False Negatives) represent the counts of positive and negative samples in the sample, while FP (False Positives) and TN (True Negatives) represent the counts of samples incorrectly predicted as positive and negative, respectively.

### 3.4 Analysis of model training time

Table 2 displays the training times for ReSinGN and SinGAN. SinGAN employs different scales when training images of various sizes. To ensure a fair comparison, ReSinGN’s training scale is set to match the scale used by SinGAN during training when generating images of different sizes. From Table 2, it can be observed that when training images are of size 125 × 125, ReSinGN trains 6.94 times faster than SinGAN. Similarly, for image sizes of 250 × 250, ReSinGN trains 7.92 times faster than SinGAN, and for image sizes of 500 × 500, ReSinGN trains 7.42 times faster than SinGAN. It is noteworthy that ReSinGN completes training for images of different sizes within minutes.

**Table 2:** Training time of ReSinGN and SinGAN.

Image size(scales)	SinGAN	ReSinGN	Speedup
125px(8)	33m27s	4m49s	× 6.94
250px(11)	60m34s	7m39s	× 7.92
500px(13)	72m33s	9m57s	× 7.42

Table 3 compares the time required for ReSinGN to generate images of different sizes when trained with two scales to the time required for SinGAN to generate images of different sizes. Experimental results (Tables 4, 5) demonstrate that ReSinGN trained with two scales can produce higher-quality images compared to SinGAN. Therefore, the actual time required to train ReSinGN to generate an image is significantly lower than the training time required for SinGAN.

**Table 3:** Comparison of training time between ReSinGN at two scales and SinGAN.

Image size(scales)	SinGAN	ReSinGN	Speedup
125px	33m27s	45 s	× 44.6
250px	60m34s	46 s	× 79
500px	72m33s	46 s	× 94.63

**Table 4:** Analysis of image quality generated by SinGAN and ReSinGN models.

	Tenengrad	RMSE	PSNR	SSIM
SiNGAN	37.1	10.4	31.1	0.80
ReSinGN	67.3	37.4	27.7	0.72

**Table 5:** Analysis of image quality generated by SinGAN and ReSinGN with different random pixel shuffle percentages.

	Tenengrad ↑	RMSE ↓	PSNR ↑	SSIM ↑
SinGAN	37.1	10.2	31.1	0.80
ReSinGN(1e-1)	35.2	11.5	31.1	0.80
ReSinGN(5e-2)	36.9	11.9	30.0	0.82
ReSinGN(1e-2)	56.3	7.6	32.6	0.91
ReSinGN(5e-3)	50.1	23.9	28.0	0.83
ReSinGN(1e-3)	54.6	32.9	27.6	0.79
ReSinGN(5e-4)	59.2	33.6	27.6	0.78
ReSinGN(1e-4)	63.9	36.0	27.7	0.73

### 3.5 Analysis of image quality generated by ReSinGN

Table 4 presents the scores obtained using Tenengrad function, RMSE, PSNR, and SSIM in Equations (7–16)were used to score the images generated by ReSinGN and those generated by SinGAN.

**Figure 13:**
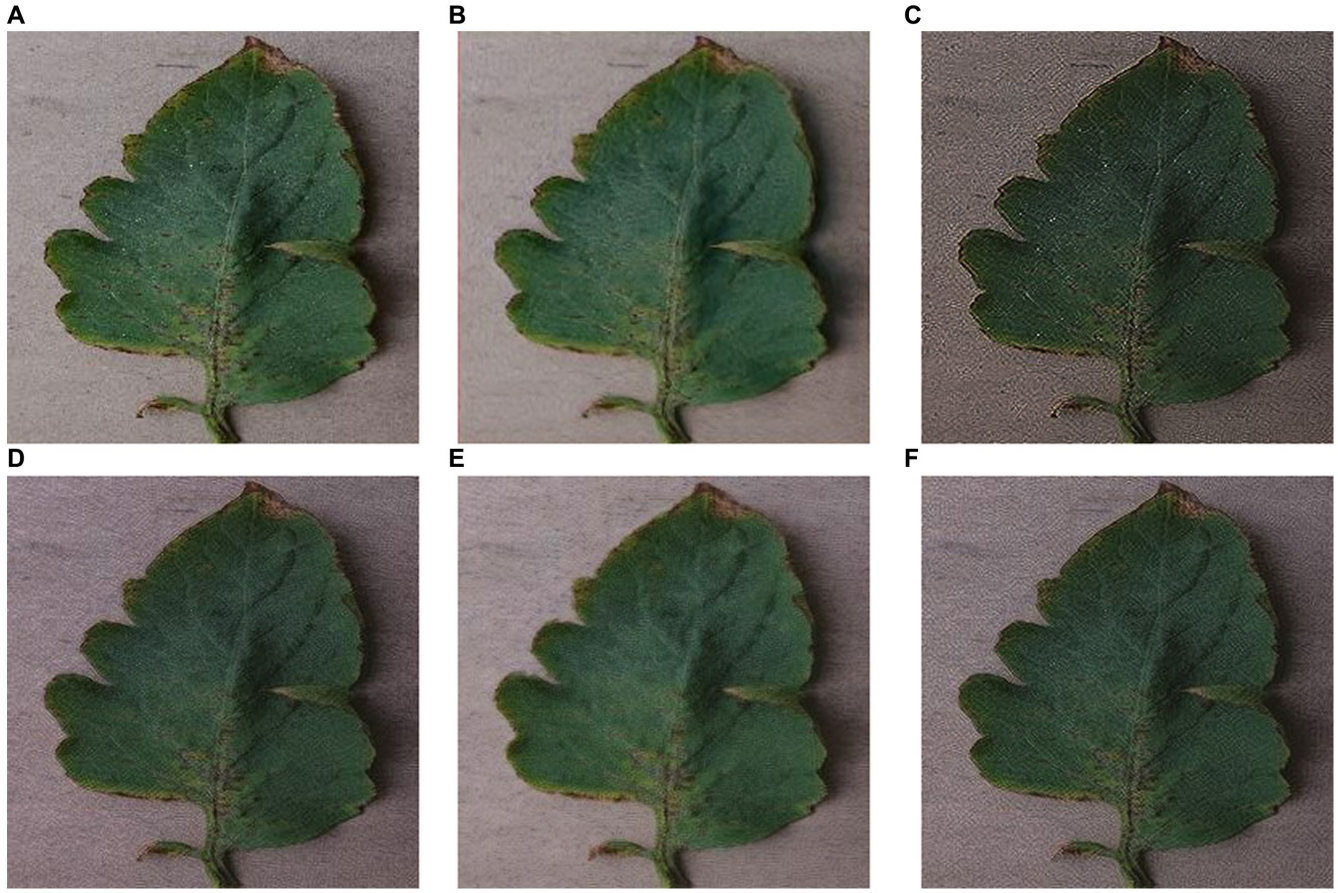
Images generated by SinGAN and ReSinGN with different random pixel shuffle percentages. **(A)** Original Image, **(B)** SinGAN, **(C)** 0, **(D)** 1e-2, **(E)** 5e-2, **(F)** 5e-3.

A higher Tenengrad score indicates clearer images, while larger PSNR, smaller RMSE, and SSIM closer to 1 indicate images that are closer to the ground truth. From Table 5, it can be observed that compared to SinGAN, ReSinGN generates images with higher clarity. However, the images generated by ReSinGN exhibit speckles, artifacts, etc. (Figure 13), resulting in lower distortion scores compared to images generated by the SinGAN. To address this issue of image distortion, this paper introduces random pixel shuffling. By adjusting the percentage of randomly shuffled pixels during training, a balance between clarity and distortion can be achieved to some extent without significantly increasing computational costs.

Table 5 compares the RMSE scores, PSNR scores, and SSIM scores of images generated by SinGAN and ReSinGN with different percentages of randomly shuffled image pixels during training. From Table 5, it can be observed that as the percentage of random pixel shuffling increases, the distortion scores of images generated by ReSinGN improve compared to SinGAN, while the clarity of the images decreases, and vice versa. When the percentage of random pixel shuffling is 1e-2, the balance between image clarity and distortion is optimized, resulting in images generated by ReSinGN that are superior to SinGAN in both clarity and image distortion.

Figure 13 illustrates the impact of the percentage of randomly shuffled pixels on the generated images. From the figure, it can be observed that when some pixels are randomly shuffled, the artifacts and speckles in the images can be effectively reduced.

To further validate the effectiveness of the ReSinGN algorithm, this paper employs ResNet34, which has a deeper network architecture, to perform a classification task on the dataset ReSinGN_7000, consisting of images generated using ReSinGN replacing a portion of the images in the original dataset, as well as on the original tomato leaf disease dataset Tomato_7000. The classification results are then compared. In this subsection, ReSinGN is trained using two scales with a scale factor of 2, random pixel transformation set to 1e-2, and a learning rate of 0.001. The specific process for substituting portions of the dataset is as follows: (1) 300 images are randomly sampled from each category of tomato leaves using simple random sampling. (2) The ReSinGN model is used to reconstruct the 300 sampled images, generating higher-quality sample images. (3) The 300 images generated by ReSinGN are then used to replace the corresponding 300 images in the original dataset for each category. Finally, the original dataset and the replaced dataset are divided into training, validation, and testing sets in a ratio of 7:2:1.

**Figure 14:**
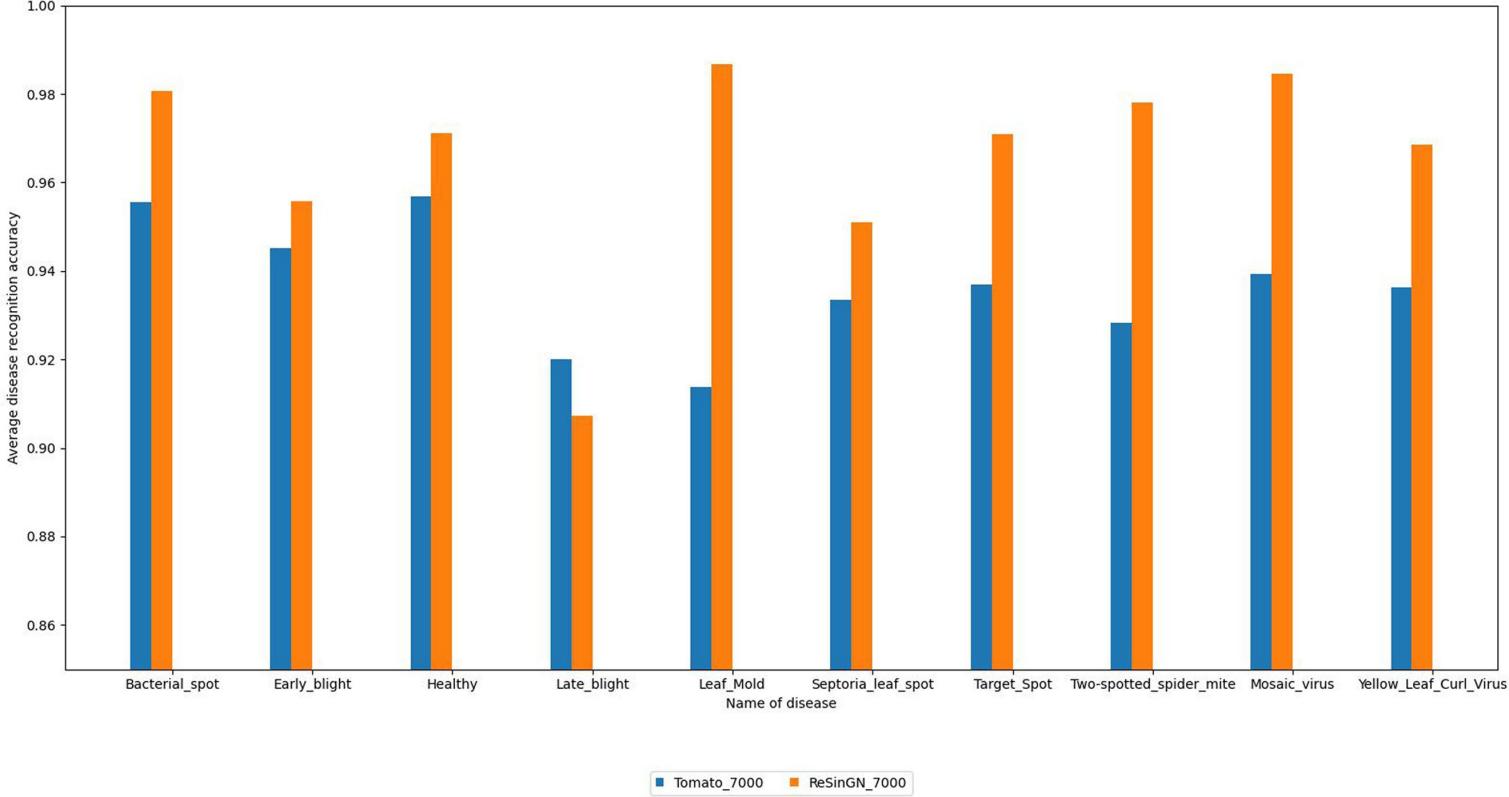
Average disease recognition accuracy of ResNet34 on different datasets.

From Table 6 and Figure 14, it can be observed that compared to the original dataset, the disease recognition accuracy, precision, recall, F1 score, and average disease recognition accuracy of the ResNet34 on ReSinGN_7000 have all been improved. There is only a slight decrease in performance for a particular disease, but the precision in identifying that disease has significantly increased. These experimental results further confirm that the images generated by ReSinGN are diverse and of higher quality.

**Table 6:** Disease recognition results of ResNet34 on different datasets.

Disease category	Dataset	Accuracy	Precision	Recall	F1 score
Bacterial_spot	Tomato_7000	0.9333	0.971	0.9571	0.964
	ReSinGN _7000	0.96	0.9855	0.9714	0.9784
Early_blight	Tomato_7000	0.8933	0.9559	0.9286	0.942
	ReSinGN _7000	0.9333	0.971	0.9571	0.964
Healthy	Tomato_7000	0.9342	0.971	0.9571	0.964
	ReSinGN _7000	0.9467	0.9714	0.9714	0.9714
Late_blight	Tomato_7000	0.8947	0.9697	0.9143	0.9412
	ReSinGN _7000	0.8933	0.9844	0.9	0.9403
Leaf_Mold	Tomato_7000	0.9067	0.9846	0.9143	0.9481
	ReSinGN _7000	0.9467	0.9853	0.9571	0.971
Septoria_leaf_spot	Tomato_7000	0.8816	0.9692	0.9	0.9333
	ReSinGN _7000	0.9333	0.9851	0.9429	0.9635
Two-spotted_spider_mite	Tomato_7000	0.8553	0.9538	0.8857	0.9185
	ReSinGN _7000	0.9067	0.9701	0.9286	0.942
Target_Spot	Tomato_7000	0.8158	0.9516	0.8429	0.8429
	ReSinGN _7000	0.96	0.9855	0.9714	0.9784
mosaic_virus	Tomato_7000	0.9211	0.9706	0.9429	0.9565
	ReSinGN _7000	0.9733	0.9857	0.9857	0.9857
Yellow_Leaf_Curl_Virus	Tomato_7000	0.8947	0.9559	0.9286	0.942
	ReSinGN _7000	0.9333	0.9577	0.9714	0.9645

### 3.6 Comparative analysis of different classification models

To validate the effectiveness of the proposed improvement strategies, the enhanced ResNet34 was compared with three classification networks through contrastive testing. The experiments encompassed the following four comparative models: ResNet34, the ResNet34-CBAM model which introduces CBAM modules into ResNet34, the ResNet34-LeakyReLU model that substitutes ReLU activation functions with LeakyReLU, and the comprehensive improved model proposed in this chapter, which incorporates both CBAM modules and LeakyReLU activation functions into ResNet34 (labeled as Ours during comparison).

**Figure 15:**
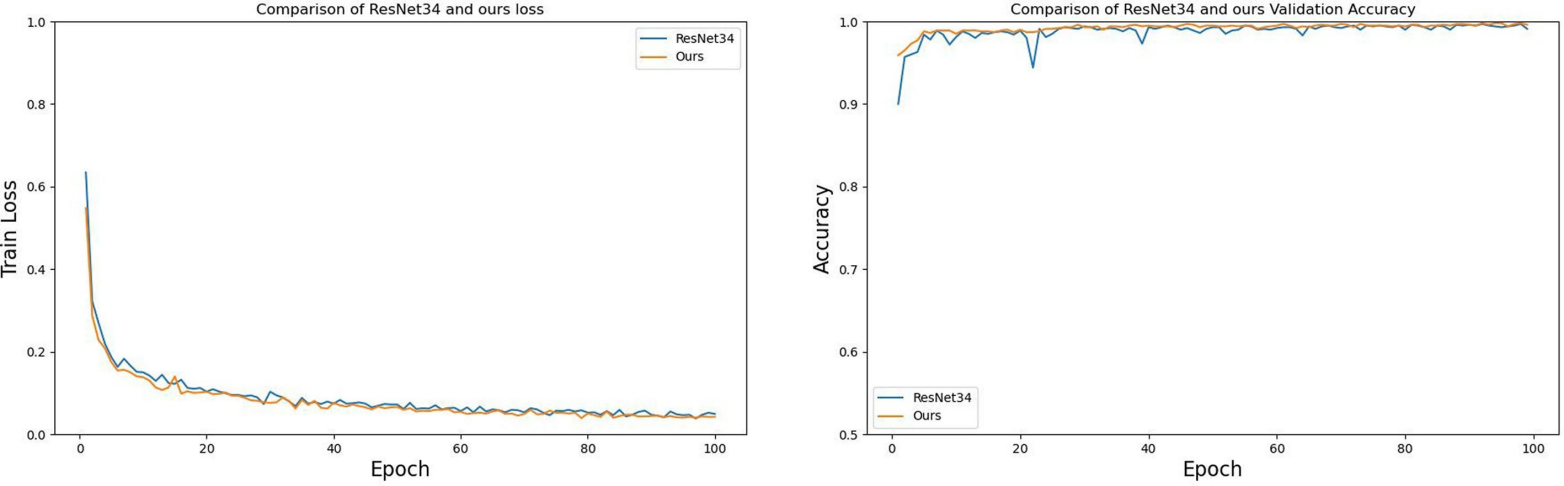
The relationship between the training loss and validation accuracy of ResNet34 and Ours as the number of epochs increases.

Figure 15 compares the relationship between the number of epochs and both training loss and validation accuracy during the training process for ResNet34 and the improved ResNet34 (labeled as Ours). From Figure 15, it is evident that: regarding training loss, the improved ResNet34 converges faster and achieves a lower final error; concerning validation accuracy, the curve for the improved ResNet34 is smoother with less fluctuation, demonstrating a more stable upward trend and consistently outperforming ResNet34. Collectively, these observations indicate that the improved ResNet34 exhibits greater stability and convergence, fits the data more closely, and possesses enhanced generalization capabilities.

**Figure 16:**
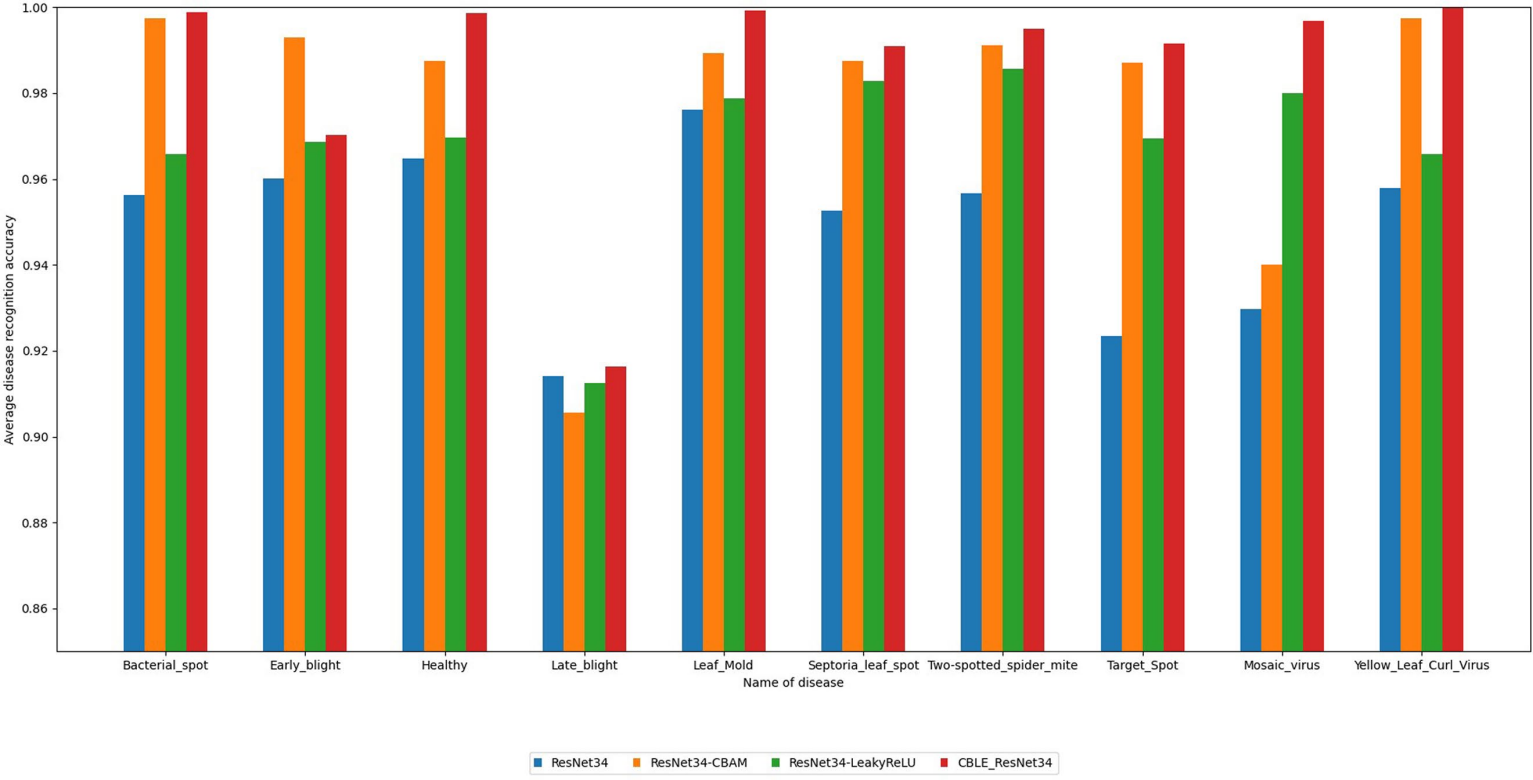
Average recognition accuracy of different models for tomato leaf diseases.

To compare the classification performance of the four networks on different types of tomato leaf diseases, this paper selects accuracy, precision, recall, F1 score, and average recognition accuracy of various leaf disease categories as evaluation metrics. The recognition results are shown in Table 7 and Figure 16.

**Table 7:** Different models’ recognition results of tomato leaf diseases.

Classification model	Accuracy	Precision	Recall	F1 score
ResNet34	0.91906	0.97803	0.936	0.95697
ResNet34- CBAM	0.9581	0.97646	0.969	0.97761
ResNet34-LeakyReLU	0.94572	0.98263	0.958	0.97095
Ours	0.96572	0.9868	0.977	0.98169

From Table 7, it can be observed that the new model constructed by combining the addition of CBAM attention mechanism and the change of activation function in ResNet34 proposed in this paper performs excellently in terms of disease recognition accuracy and average recognition accuracy of tomato leaf diseases, reaching 98.68 and 98.57% respectively, which are significantly higher than the original ResNet34 (97.8, 94.92%). From Figure 16, it is observed that the algorithm proposed in this paper achieves higher average recognition accuracy for various categories of tomato leaf diseases compared to the original ResNet34. It is noteworthy that compared to the other three models, the proposed algorithm achieves the highest recognition accuracy for the nine categories of tomato leaf diseases, and ranks second only in the average recognition accuracy for one category of tomato leaf disease. In summary, the proposed method demonstrates superior performance in tomato leaf disease recognition tasks.

## 4 Discussion

This paper aims to improve the recognition rate of tomato leaf diseases by proposing a data augmentation model based on improved SinGAN and a disease recognition model based on improved ResNet34 to assist in intelligent recognition of tomato leaf disease images. The key focuses of this research are as follows:

1. Addressing the issues of long training time and potential image distortion in SinGAN, a single-image generation network based on reconstruction, named ReSinGN, is proposed. ReSinGN replaces the GAN in SinGAN with an autoencoder equipped with a CBAM module, making the training objective image reconstruction, which simplifies the learning task. Additionally, random pixel transformations are introduced in ReSinGN to control the trade-off between clarity and distortion to some extent by adjusting the percentage of randomly shuffled pixels during training. Experimental results demonstrate that: (1) ReSinGN has significantly shorter training time compared to SinGAN while generating clearer images; (2) When the percentage of random pixel transformations is set to 1e-2, the ReSinGN model achieves an optimal balance between image clarity and distortion, producing images superior to SinGAN in both clarity and distortion aspects. In summary, the use of ReSinGN results in images of better quality and greater diversity.
2. ResNet34 was further employed to validate the effectiveness of ReSinGN. Partial data samples in the original tomato leaf disease dataset were replaced with data generated by ReSinGN, and ResNet34 was used to classify the original dataset and the replaced dataset for comparison. The classification experimental results indicate that the disease recognition accuracy, precision, recall, F1 score, and the average disease recognition accuracy of ResNet34 on the replaced tomato leaf dataset have been improved by 4.56, 1.28, 3.86, 2.56, and 2.89%, respectively.
3. A plant leaf disease identification method based on ReSinGN and improved ResNet34 is proposed. Firstly, the tomato leaf disease dataset is augmented using the ReSinGN model to enhance the overall quality of the dataset. Secondly, a Convolutional Block Attention Module (CBAM) is introduced to dynamically adjust the attention weights of each position in the ResNet34, thereby partially addressing the issues of insufficient local feature integration and parameter sharing in the network, leading to improved performance. Then, the ReLU activation function is replaced with the LeakyReLU activation function to prevent neuron death. Finally, a training method based on transfer learning is employed to accelerate the network training process. Experimental results demonstrate that the improved ResNet34 achieves an average recognition accuracy and precision of 98.6 and 98.68%, respectively, for tomato leaf disease, validating the effectiveness of the proposed improvements. This provides an automated solution for the prevention and control of tomato leaf diseases.

## 5 Conclusion

The innovation of this paper lies in applying the ReSinGN model to data augmentation of tomato leaf disease images and using an improved ResNet34 for disease recognition, achieving certain results. However, there are still some shortcomings: (1) The ReSinGN model proposed in this paper trains significantly faster than SinGAN and produces images of higher quality. Future research could explore extending this approach to the domain of multi-image training. (2) Although the improved ResNet34 in this paper has achieved excellent results in tomato leaf disease recognition tasks, with an average recognition accuracy of 98.6%, it ranks only second in the recognition accuracy of one of the diseases. Therefore, it is necessary to explore other methods to further enhance the model’s performance.

## Data availability statement

The original contributions presented in the study are included in the article/supplementary material, further inquiries can be directed to the corresponding author.

## Author contributions

JC: Writing – original draft, Software, Methodology, Conceptualization. HH: Writing – original draft, Visualization. JY: Writing – review & editing, Resources, Funding acquisition.
